# Sustaining Intravitreal Residence With L-Arginine Peptide-Conjugated Nanocarriers

**DOI:** 10.1167/iovs.17-22160

**Published:** 2017-10

**Authors:** Hao Li, Wenzhong Liu, Christine M. Sorenson, Nader Sheibani, Daniel M. Albert, Thulani Senanayake, Serguei Vinogradov, Jack Henkin, Hao F. Zhang

**Affiliations:** 1 Department of Biomedical Engineering, Northwestern University, Evanston, Illinois, United States; 2 Department of Pediatrics, University of Wisconsin-Madison, Madison, Wisconsin, United States; 3 Departments of Ophthalmology and Visual Sciences, Biomedical Engineering, and Cell and Regenerative Biology, University of Wisconsin-Madison, Madison, Wisconsin, United States; 4 Department of Ophthalmology, Casey Eye Institute, Oregon Health Sciences University, Portland, Oregon, United States; 5 Department of Pharmaceutical Sciences, College of Pharmacy, University of Nebraska Medical Center, Omaha, Nebraska, United States; 6 Center for Developmental Therapeutics, Northwestern University, Evanston, Illinois, United States; 7 Department of Ophthalmology, Northwestern University, Chicago, Illinois, United States

**Keywords:** intravitreal drug delivery, nanoparticle, fluorescence imaging

## Abstract

**Purpose:**

Intravitreal injection of antiangiogenic agents is becoming a standard treatment for neovascular retinal diseases. Sustained release of therapeutics by injecting colloidal carriers is a promising approach to reduce the injection frequency, which reduces treatment burdens and the risk of complications on patients. Such sustained release often requires carriers to have micrometer-scale dimension that, however, can potentially promote glaucoma and inflammation. Small, polycationic particles can be immobilized in vitreous through multiple cooperative ionic interactions with hyaluronic acid of the vitreous interior, but such particles are generally toxic. Here, we synthesized and examined a biocompatible dextran-based nanocarrier (<50 nm in diameter) conjugated with cationic peptides containing L-arginine with minimal toxicity, aiming to provide sustained release of therapeutic drugs in vitreous.

**Methods:**

We synthesized the nanocarriers with condensed cholesteryl dextran (CDEX) as core material. Cationic peptides containing 1 to 4 arginine groups, along with fluorescence tags, were conjugated to the CDEX surface. We monitored the carrier diffusion rate ex vivo and half-lives in vivo in rodent vitreous using fluorescence imaging. We evaluated the toxicity by histological examinations at the second, third, eighth, and thirty-sixth week.

**Results:**

The diffusion rate of nanocarriers was inversely related to zeta potential values in freshly isolated vitreous humor. We observed increased half-lives in vivo with increasing zeta potential (up to 240 days). Histological examinations confirmed no adverse effects on ocular morphology and organization.

**Conclusions:**

We demonstrated the potential of L-arginine peptide-conjugated nanocarriers toward safe and sustained therapeutic release system for posterior eye diseases.

Retinal and posterior segment diseases affect nearly 10 million people in the United States.^1^ If left untreated, they could result in loss of vision and diminished quality of life.^2,3^ The effective treatments largely rely on a safe and efficient mode of therapeutic drug delivery. The unique anatomic and physiologic barriers of the eye block outside drug molecules from entering the posterior segment, thus leaving the delivery efficiencies of conventional topical or systemic/oral drug administrations to be less than 5%.^4–6^ Currently, intravitreal injection is the most efficient method for posterior eye drug delivery.^7^ This method directly delivers active agents near the lesions, increasing the local drug concentration with low systemic exposure. Intravitreal injection is the primary method to treat endophthalmitis, submacular/vitreous hemorrhage, retinal vascular occlusion, advanced AMD, and diabetic retinopathy.^8,9^ In recent years, the acceptance of intravitreal injections has grown rapidly because of the injection of VEGF inhibitors, which slows down the progress of neovascular retinal diseases, such as exudative AMD and diabetic retinopathy.^7,10^ In 2012, more than 2.3 million injections were reported, and the number was estimated to reach 6 million in 2016.^11^

Despite many advantages and the increase in acceptance of intravitreal injections, patients will endure a burden of frequent injections over the long-lasting treatment period.^12^ Most therapeutic agents are typically eliminated from vitreous in a short time following their administration.^13^ Consequently, repeated injections are needed to maintain a therapeutically effective concentration in the posterior eye. For example, the half-life of ranibizumab (a VEGF inhibitor for treatment of exudative AMD) in human vitreous is approximately 9 days, requiring one administration per month.^14,15^ Frequent intravitreal injections raise the patient's discomfort, and cumulatively increase the risk of potential complications, such as vitreous hemorrhage, cataract, and endophthalmitis.^16^ Additionally, frequent outpatient visits are a burden for vision-impaired patients and significantly increase total treatment cost.^12,17^

In the past few years, there have been many efforts to overcome the shortcomings of multiple injections by extending the time of therapeutic release after delivery.^5^ Among various proposed approaches, the slow-released colloidal drug carrier has become a preferred approach.^5,12,13,17^ The colloidal drug carriers are normally nano/microspheres made of biodegradable materials, with drug molecules embedded in their body or coated on their surface. After injection, the carriers remain in the vitreous over a relatively long period and slowly release the therapeutic molecules during their breakdown to smaller fragments until total clearance.^17^ The advantages of colloidal carriers include biocompatible material for the carrier, minimally invasive administrations, and minimal complications after well-established intravitreal injection procedures.

One critical issue limiting the clinical uses of the colloidal carriers is their relatively large size. Colloidal carriers generally rely on large particle size (larger than 1 μm) to prolong the diffusion through the viscous vitreous. In 2001, Sakurai et al.^18^ studied the dynamics of fluorescent polystyrene particles with diameters between 0.05 μm and 2 μm in vitreous. Particles approximately 50 to 200 nm moved to the retina, whereas 2-μm particles remained in vitreous. In 2013, Shmueli et al.^13^ injected particles approximately 6 to 12 μm in mouse eyes and achieved slow particle degradation in vitreous over 200 days, potentially reducing the injection frequency to once per year. However, large carriers are unstable to autoclaving, and not amenable to sterile filtration, they or their breakdown products with a continuum of intermediate sizes may block vision,^19^ and, more importantly, may potentially lodge in or interact with the trabecular drainage meshwork and its resident phagocytes, causing glaucoma or inflammation.^20^

One potential solution to reduce particle size while maintaining slow diffusion is to use cationic nanoparticle carriers. The vitreous has a very high content of polyanionic polymers, especially the viscosity-enhancing hyaluronic acid (HA). Particles with polycationic compositions will anchor themselves to HA in vitreous through ionic binding, resulting in slow diffusion. In 2009, Kim et al.^21^ showed that albumin, freely diffusing in vitreous, was immobilized by converting its surface acidic residues to cationic amides with ethylenediamine (zeta potential change from −33 to + 11.7 mV). In 2013, Xu et al.^12^ reported that 200-nm polystyrene particles coated with cationic amine groups showed diffusion rates of 1000-fold slower than their neutral or anionic counterparts in bovine vitreous. Using such cationic carriers, however, was not clinically practical because the particles are conjugated with multiple amino groups, which, along with most other cationic groups, are toxic, especially when multiple cations are on the particles of a size that can be engulfed by cells.^22^

In 2011, Zern et al.^23^ reported that when the positive charge is derived exclusively from L-arginine (L-Arg), small cationic particles were at least two orders of magnitude less toxic to cells. They compared cytotoxicity among cationic nanoparticles with varied sources of positive charges, including polyethylene imine (PEI), polyamidoamine polyvinyl pyridinium bromide (PAMAM) dendrimers, L-Arg, and D-arginine (D-Arg).^23^ They found that although all the particles are capable of forming complexes with polyanions, the L-Arg–based carriers were at least 200-fold less toxic than the non–arginine-based cationic carriers and at least 10-fold less toxic than D-Arg carriers. Supporting evidence was then reported by Veiseh et al.,^24^ who compared the safety of cationic magnetic nanovectors coated with either PEI or L-Arg. L-Arg particles showed no toxicity when injected into mice, whereas the PEI-based carriers produced hemolysis, erythrocyte aggregation, and acute organ toxicity. Sarker et al.^25^ compared lipofectamine transfection with 100-nm-diameter liposomes based on inserted lipid L-Arg. Those with zeta potentials higher than 20 mV gave far superior DNA delivery and displayed very low toxicity. The nontoxicity of L-Arg coating was further confirmed with dendrimers both in vitro and in vivo in delivery of Hsp27 small-interfering RNA to prostate tumor cells, this was efficacious against tumor growth in mice.^26^ This range of observations suggested that L-Arg could be a practical and safe cationic group for anchoring nanoparticle carriers in vitreous humor.

Based on these findings, we designed and fabricated a novel L-Arg peptide-based cationic nanoparticle, aiming to establish nontoxic, biocompatible therapeutic carriers for ocular drug delivery. Our nanoparticles were smaller than 50 nm in diameter, made from the neutral polysaccharide, dextran, with L-Arg–containing peptides covalently linked to provide nontoxic positive charges for vitreous anchoring. We experimentally confirmed ionic binding as the mechanism of slowed particle diffusion in a charge-dependent manner, measured particle diffusion rate ex vivo, and monitored the half-life in vivo in rat vitreous, and their relation to surface charge of the carriers (zeta potential). We further evaluated the adverse effects of fabricated nanoparticles on ocular integrity by histological examination.

## Materials and Methods

### Nanoparticle Carrier Design

**Figure 1:**
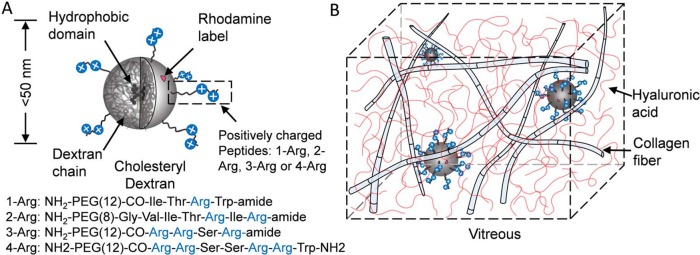
Schematic of the nanoparticles and drug delivery mechanism. (**A**) Particle structure. CDEX nanoparticle (less than 50 nm in diameter). Hydrophobic domains are inside the particle close to the core. Particles were labeled by rhodamine B with less than three molecules per particle. Three types of positively charged peptides were linked on the particle surface, with a density of 19 to 30 peptides per particle. (**B**) Particles are smaller than the vitreous fiber mesh. They are trapped in vitreous by ionic binding between peptides on particle surface and HA coils.

An illustration of the nanoparticle structure is shown in Figure 1A. We chose condensed clusters of cholesteryl dextran (CDEX; 5 mol %, hydrophobic domain is presumed near the nanoparticle center), forming its core material. Dextran is a biocompatible compound widely used in Food and Drug Administration–approved plasma expanders^27,28^ and ocular products.^29^ It can form compact spherical nanoparticle carriers with a range of molecular weight (and size).^30^ Also, CDEX has been ester-linked to decanoate, demonstrating drug delivery potential of condensed forms with release of small synthetic compounds from the nanoparticle hydrophobic reservoir, accelerated by added esterases.^31^ In this work, we chose the CDEX nanoparticles smaller than the pore size of trabecular meshwork and vitreous collagen fiber meshwork (less than 50 nm).

Cationic peptides providing anchorage were covalently attached to the CDEX particle surface through carbamate bonds, after activation of OH groups of sugar units in CDEX. The peptides were designed to contain naturally occurring amino acid sequences from proteins commonly found in plasma or extracellular matrix to minimize toxicity and immunogenicity. Specifically, four distinct amino-PEG(8–12)-peptides, containing four to seven total amino acids providing positive charges exclusively from one to four L-Arg groups, were conjugated covalently to the surface. (As described later, the four L-Arg peptide conjugate, with zeta potential of 9 to 10 mV, was highly immobilized in vitreous humor, and its diffusion was not further studied.) We chose PEG-peptides of approximately 50 to 75 Å in total length, with 20 to 30 Å of cationic groups linked with approximately 30 to 45 Å of N-terminal amino-PEG groups (8,12 EG units). The L-Arg groups would thus be at the distances of 35 to 60 Å extended from the particle surface. We found 19 to 30 peptides were conjugated to one CDEX particle (assuming CDEX particle molecular weight [MW] to be 77 kDa, before conjugation).

Such conjugation has only a modest 20 to 25 wt.% effect on overall particle mass, but enables arginine cations to move freely at the particle surface to access and pair with anionic units of HA and similar polymers. Rhodamine (Rh) B chromophore was also covalently conjugated to the particle surface with a fluorophore load of less than three chromophores per particle as a tag for imaging.

The proposed mechanism of the long-lasting nanoparticles in vitreous is shown in Figure 1B. Vitreous humor is a transparent gel-like substance containing 99% water, with highly cross-linked collagen fiber–HA network to maintain the shape. HA molecules are anionic and in a random coil structure. HA fills the space between collagen fibers to prevent aggregation. Our nanoparticles are not trapped by the collagen fiber network due to their small size, rather are immobilized by the sum of multiple weak ionic associations between the peptide L-Arg residues anchored on the particle surface (blue circles) and HA carboxylate groups (red strands).

### Nanoparticle Carrier Synthesis

BOC-amino-PEG(n)-carboxylic acids were obtained from Quanta Biosciences (Beverly, MA, USA). Rh-isocyanate was from Thermo Fisher (Waltham, MA, USA). Ethanolamine, 1,1′-carbonyldiimidazole (CDI), cholesteryl chloroformate, mono-BOC-ethylenediamine, 2,2′-(ethylenedioxy) bis (ethylamine) and dextran (Leuconostoc, 70 kDa) were from Sigma Aldrich (St. Louis, MI, USA). Amino-rhodamine (a-Rh) was formed by reaction of Rh-isocyanate with excess mono-BOC-ethylenediamine. It was purified by silica gel chromatography (CH_2_Cl_2_: MeOH, 4:1). BOC was removed with 30% trifluoroacetic acid (TFA) in dichloromethane.

CDEX gel was formed by adding 0.3 g cholesterol chloroformate and one equivalent of triethylamine in 8 mL dichloromethane into 45 mL anhydrous stirred DMSO containing 3.0 g dry dextran, reacting at 40°C for 3 hours. After quenching with 45 mL water, the mixture was dialyzed exhaustively against water, and freeze dried. The crude product was re-dissolved in 50 mL water and sonicated (Branson bath, Danbury, CT, USA) for 2 hours. The solution was then filtered through 0.8 μm, 0.45 μm, and 0.2 μm syringe filters consecutively to remove suspended material before final lyophilization.

In the synthesis of the CDEX, cholesterol chloroformate covalently attaches cholesterol units to 70-kDa dextran as di-carbonate linked units. With approximately 370 glucose units per dextran particle, 16 to 20 cholesterol molecules will be appended, most of them self-associate to form a hydrophobic condensed core. This contracts the dextran into a smaller compact nanoparticle with high water solubility, displaying more than 100 sugar OH groups on their surface.^32^ Those surface hydroxyl groups can be conjugated to multiply hydrophilic drugs or targeting agents such as proteins or peptides.^33,34^ Here we use such conjugation to cationic peptides to enhance residence time of nanoparticles in the vitreous humor.

Four peptides for conjugation were synthesized by solid state methods with fluorenylmethyloxycarbonyl coupling methods on Rink amide resin, with final coupling to BOC-PEG-acid, and resin release and de-blocking with TFA. Each peptide contains an amino-PEG(n)CO- cap at its N-terminus, where n is 8 or 12 ethylene glycol units, as extenders. The structures for peptides containing one to four L-Arg groups (1-Arg, 2-Arg, 3-Arg, and 4-Arg) are shown in Figure 1A. All were purified by preparative reverse-phase C18 HPLC (>95%), isolated as TFA salts.

CDEX was activated for aminopeptide coupling by CDI reaction. CDEX (210 mg, 3.0 μmol) was dried by vacuum evaporation from anhydrous pyridine (2 × 5 mL), then dissolved in 10 mL anhydrous DMSO. Freshly opened CDI (36 mg; 220 μmol) solution was added dropwise and stirred for 4 hours at 40°C. The CDEX-Im thus formed was dialyzed against water overnight at 4°C and lyophilized, giving 169 mg product. Nuclear magnetic resonance estimated 58 imidazole groups attached per particle (assuming one 70-kDa dextran molecule forms one CDEX particle).

Peptides were coupled as carbamate to the above CDEX-Im particles as follows: 5 mg of the above CDEX-Im (3.5 μmol imidazolyl) was dissolved in 0.5 mL water, and its pH value was adjusted to 8.0 with NaHCO_3_. Peptide (3.5 μmol) dissolved in 0.2 mL of dimethylformamide (DMF) was stirred into the CDEX-Im solution and reacted overnight at 25°C, after which excess Im- of active groups was quenched by additional overnight reaction with 4 μL (50 μmol) ethanolamine. For each batch, a parallel batch was first reacted overnight with a-Rh. After peptide coupling and ethanolamine quenching, conjugates were exhaustively dialyzed against water at 4°C, then lyophilized. The product was re-dissolved in water to a concentration of approximately 0.4 mg/mL and then sterile filtered through a 0.2-μm syringe filter (see Supplementary Materials for synthesis schemes).

The parallel batch described above for fluorescence monitoring was prepared as follows: a small tag of Rh (approximately 2.0–2.5 μmol dye per 100 mg of CDEX) was attached to the same conjugates prepared in parallel reaction after initiation of peptide coupling. A-Rh as the fluorescence tag was synthesized by modification of Rh isothiocyanate with a 3-fold excess of 2,2′-(ethylenedioxy) bis (ethylamine) and purified by column chromatography on silica gel using a stepwise gradient of methanol in dichloromethane. Before tagging, the carbonylimidazole-activated CDEX (5 mg in 0.5 mL water with pH adjusted to 8) was reacted with 3.5 μmoles peptide dissolved in 0.2 mL DMF (stirred at 25°C). The above a-Rh (0.1–0.2 mg) was then added, and the reaction was continued at 25°C for 24 hours. Reaction mixture was then quenched with 4 μL ethanolamine and left overnight at 4°C. Fluorescent CDEX-peptide conjugate was purified by dialysis in semipermeable membrane tubes (MW cut-off 3500) against water at 4°C under stirring overnight and was then freeze dried (total yield: 64%–86%). The concentration of Rh tags was typically 15 to 25 μM in conjugates in aqueous solution at 1 mg/mL concentration (8–11 μM particles, measured by spectrophotometry). The tagged nanoparticle solution was prepared to 0.4 mg/mL and mixed with their untagged counterpart conjugates (also 0.4 mg/mL) to reduce the Rh tag concentration to 1 to 2 μM in the solution for ex vivo and in vivo nanoparticle monitoring.

The carbamate bonds linking both peptides and fluorescent tag to CDEX are well-established drug functional groups in drug development. They are common structural elements of many approved drug agents, often substituted for amide bonds as carbamates are slowly hydrolyzed by peptidases.^35^ These bonds are better known in polymer coating chemistry such as in urethanes. Block copolymers of urethanes (carbamates) with PEG formed thermal gels, and have been used to deliver tethered peptides.^36^ They are normally stable for many months under physiologic conditions, and are resistant to aqueous breakdown below 55°C.^37^ They can be hydrolyzed by chymotrypsin and esterases, but vitreous humor is expected to have very low content of such enzymes^38^ (See Supplementary Materials for illustration of peptide and fluorescence tag link).

### Characterization of Physical and Chemical Properties

Quantification of average peptide load was accomplished by weighing a portion of each exhaustively dialyzed and lyophilized conjugate, followed by aqueous dissolution. Peptide concentration was then determined by means of a BCA peptide/protein assay calibrated against a standard curve of the free peptide, which gave a higher accuracy than nuclear magnetic resonance method. With conjugated peptide molarity, [P] from BCA assay, the peptide load, Lp, can be calculated by Lp = [P]/[CDEX], where [CDEX] is the CDEX molarity. To calculate [CDEX], we first weighted the dried conjugate per milliliter. We then obtained the weight contribution from CDEX by subtracting the weight of conjugated peptides, which equals to [P] multiplied by peptide MW. Finally, we calculated [CDEX] by dividing the weight contribution of CDEX per milliliter by CDEX MW. In the calculation, peptide MW was based on the amino acid and PEG composition, which also included the bicarbonate counter ion mass per arginine residue. In our work, Lp was calculated to be 19 to 30 peptides per particle Because CDEX made from 70-kDa dextran had an average MW of 77 kDa, and the peptides had typical MWs near 1000 to 1200 g/mole, the final products had average MWs of approximately 90 to 105 kDa.

Zeta potential and size were measured on non-Rh product in PBS using electrophoretic light scattering and dynamic light scattering, respectively (Zetasizer ZS90; Malvern, UK). The Z averaged particle size was 15 nm and the full width at half maximum of the distribution peak was 24 nm. Zeta potentials for 1-Arg, 2-Arg, 3-Arg, and 4-Arg are 0.07 mV, 2.2 mV, 4.6 mV, and 9.2 mV, respectively.

### Ex Vivo Diffusion Rate Measurements in Rodent Vitreous

#### Preparation of Fresh, Intact Rat Vitreous Gels

Rat (Long-Evans; Charles River, Wilmington, MA, USA) eyeballs were enucleated immediately after euthanasia. We carefully removed conjunctive tissues and placed the eyeballs in a homemade concave holder (6 mm in diameter) with pupil facing upward. Vitreous body is very fragile. Vitreous collagen fibers are tightly linked to both the anterior segment and retina tissues.^39^ Vitreous tends to liquefy when removed from the eyeball due to collapse of collagen fibers.^12,39^ To maximally preserve the structure of vitreous, we removed only the cornea and iris for monitoring. We first made a small incision at the corneal limbus using a scalpel blade, circumferentially cut along the limbus using a scissor, and separated cornea from sclera. We then carefully picked out iris using a small tweezer. To cancel the dioptric power of the ocular lens, we added a coverslip on top of the opened globe. A tiny amount of water was added between the coverslip and ocular lens for refractive index matching. A ring spacer was used to hold the coverslip from pressing the globe (see Supplementary Materials).

#### Diffusion Rate Measurements

The opened globe with the coverslip was placed under a stereo microscope (SMZ1500; Nikon, Tokyo, Japan) equipped with a high-sensitive charge-coupled device (PixelFly qe; PCO AG, Kelheim, Germany) and a long-pass optical filter (FEL0550, cut-off wavelength: 550 nm; Thorlabs, Newton, NJ, USA) for fluorescence imaging (see Supplementary Materials). Two microliters of nanoparticle colloidal gel was injected to the center of vitreous by a microliter syringe (30G needle; Hamilton, Reno, NV, USA). Nanoparticles carrying four different peptides were separately injected and monitored to determine the influence of various peptides' zeta potential on the rate of diffusion.

We excited Rh labels using a continuous-wave 532-nm laser (10 mW) and recorded the fluorescence distribution every 5 minutes. Because the rat ocular lens occupies almost two-thirds of the rat eyeball volume, rat vitreous body is in a crescent shape and could be roughly considered as a two-dimensional structure.^40^ Therefore, we estimated the fluorescence diffusion area (edge defined as *e*^−1^ of the center fluorescence intensity) at each time point, and numerically fitted the area using a two-dimensional model to calculate the diffusion coefficient^41^ (see Supplementary Materials). For each measurement, the preparation and monitoring were completed within 3 hours. The eyeballs were stored in ice during transportation, and recovered to room temperature before injection and monitoring.

### In Vivo Half-Life Measurements

#### Intravitreal Injection

Adult rats (250-g Sprague Dawley; Charles River Laboratories) were used for in vivo measurements. Before intravitreal injection, animals were anesthetized by an intraperitoneal injection of a mixture of ketamine and xylazine (ketamine: 11.45 mg/mL; xylazine: 1.7 mg/mL, in saline; 10 mL/kg body weight).

We used a microliter syringe with a 30G needle (Hamilton) to perform intravitreal injections. The anesthetized rats were placed under a stereomicroscope. We inserted the needle to the posterior chamber from limbus, and held the syringe still. We pushed the syringe until solution was completely injected to the posterior chamber, and then gently withdrew the needle. Lubricate ophthalmic ointment was applied to eyes after injection.

We intravitreally injected nanoparticle carriers loaded with the cationic peptides of 1-Arg (*n* = 6), 2-Arg (*n* = 5), 3-Arg (*n* = 3), and 4-Arg (*n* = 3) to study the relationship between nanoparticle zeta potential and its half-life. The injection dose was 1.5 μL, approximately 2.7% of a rat's total vitreous volume.^42^ PBS of the same volume was injected in the control group (*n* = 3).

#### Fundus and Fluorescence Imaging

Fundus and fluorescence images were taken every 3 days in the following week after injection and then every 7 days after the first week. Animals were anesthetized by a mixture of isoflurane and air (2% isoflurane at 3 L/min for 10 minutes and 1.5% at 2 L/min in following experiments). The rat eyes were anesthetized using a drop of 0.5% tetracaine hydrochloride ophthalmic solution and dilated using a drop of 1% Tropicamide ophthalmic solution. During imaging, animals were placed on a homemade animal holder. Artificial tears were applied every 2 minutes to keep the cornea moist.

We took fundus reflectance images to locate the region of interest through retinal landmarks, and took a fluorescence image immediately thereafter using a customized high-resolution rodent fundus camera.^43^ The reflectance fundus images were taken using a narrow spectral-band illumination (halogen lamp with band-pass filter; bandwidth: 12.7 nm, center wavelength: 580 nm) to minimize chromatic aberrations. For fluorescence imaging, a 532-nm continuous-wave laser was combined into illumination optical path by a 45-degree laser-line mirror. A 550-nm long-pass filter (Edmund Optics, Barrington, NJ, USA) was added before the camera to block the excitation light, and let the fluorescence and reflected light to pass. The system's optical resolution was 10 μm. The imaging field of view was 50 degrees. By adjusting the fundus imager, peripheral retinal area also can be monitored (see Supplementary Materials). The power of the illumination light and the laser excitation was 0.2 mW and 0.25 mW, respectively, which were below the ANSI ocular laser safety limit.^44,45^ The exposure times were 0.2 second for fundus imaging and 2 seconds for fluorescence imaging. All experiments were performed in compliance with the ARVO Statement for the Use of Animals in Ophthalmic and Vision Research and were approved by the Animal Care and Use Committee of Northwestern University.

### Histological Examination

Rats with no fluorescence detected for 2 weeks, or 36 weeks after intravitreal injection, were euthanized, eye globes were enucleated, and immediately fixed in formalin and prepared for histological evaluations. Paraffin sections were stained with hematoxylin-eosin and examined for structural abnormalities and signs of inflammatory infiltrations in masked fashion.

## Results

### Ex Vivo Diffusion Rate in Vitreous

**Figure 2:**
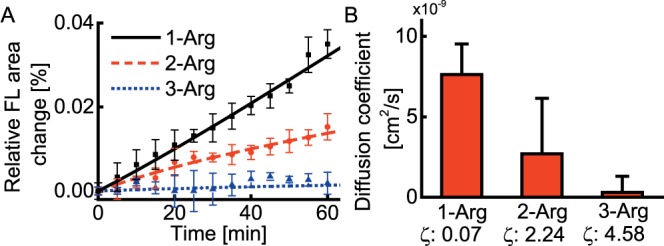
Ex vivo diffusion coefficient measurements in rat vitreous (*n* = 9). (**A**) Relative fluorescence area change as a function of time. (**B**) Diffusion coefficient estimated from (**A**). FL, fluorescence; ζ, zeta potential, unit mV.

The nanoparticle diffusions with time are shown in Figure 2A and the calculated diffusion rates are shown in Figure 2B. Diffusion rates of 4-Arg particles are not shown due to the difficulties of measuring the extremely slow particle movement during the observation period. To minimize the influence from initial injection conditions, we used relative area changes to calculate diffusion rates (see Supplementary Materials).

Nanoparticle surface modified with peptides containing 1-, 2-, or 3-Arg groups had distinct diffusion behaviors. Nanoparticles modified with 1-Arg peptides expanded the fluorescent area quickly, 3-Arg peptide modified nanoparticles diffused only minimally in 1 hour, and the diffusion of 2-Arg peptide modified nanoparticles was intermediate. The calculated diffusion coefficients for 1-Arg, 2-Arg, or 3-Arg peptide modified nanoparticles were 7.6 × 10^–9^ cm^2^/s, 2.7 × 10^–9^ cm^2^/s, and 3.0 × 10^–10^ cm^2^/s, respectively. The diffusion coefficient for 3-Arg peptide modified nanoparticles could be within the measurement precision, because the nanoparticles barely diffused in 1 hour. Compared with the theoretical diffusion coefficient of uncharged particles, the diffusion coefficients of the fabricated nanoparticles were significantly reduced. The diffusion coefficient of 50-nm nanoparticles in water is approximately 1 × 10^−7^ cm^2^/s.^41^ Considering the viscosity of vitreous as four times that of water,^39^ this number in the vitreous should be 2.5 × 10^−8^ cm^2^/s. Compared with this value, 1-Arg modified nanoparticle had only a very modest effect in reducing the diffusion rate of the carriers by approximately 4-fold, whereas the diffusion coefficients of 2-Arg and 3-Arg nanoparticles were significantly reduced by one and two orders of magnitude. The 2-Arg nanoparticles were comparable to 450-nm-diameter particles, and 3-Arg nanoparticles were comparable to micron-scale particles in terms of diffusion coefficient. The diffusion coefficients were inversely proportional to the zeta potential of peptide modified nanoparticles, suggesting that the ionic interaction is responsible for the reduced particle diffusion, assuming dye labeling did not affect the diffusion due to their tiny amount.

We further demonstrated the ionic binding between nanoparticles and HA molecules by competitive binding (see Supplementary Materials). We injected the mixture of the nanoparticles and protein molecules with a high zeta potential (protamine) to the vitreous ex vivo, and monitored the particle diffusion. Protein molecules carrying positive charge can competitively bind to HA. Such competitive binding will increase the nanoparticles' diffusion if nanoparticles are also ionically bound to HA, but will minimally affect the diffusion if the reduced diffusion was caused by the trapping of collagen fiber network in our previous experiments. In the competition binding assay, we observed significant increases in the nanoparticle diffusion, confirming the ionic biding between the nanoparticles and HA.

### In Vivo Half-Life Measurement

**Figure 3:**
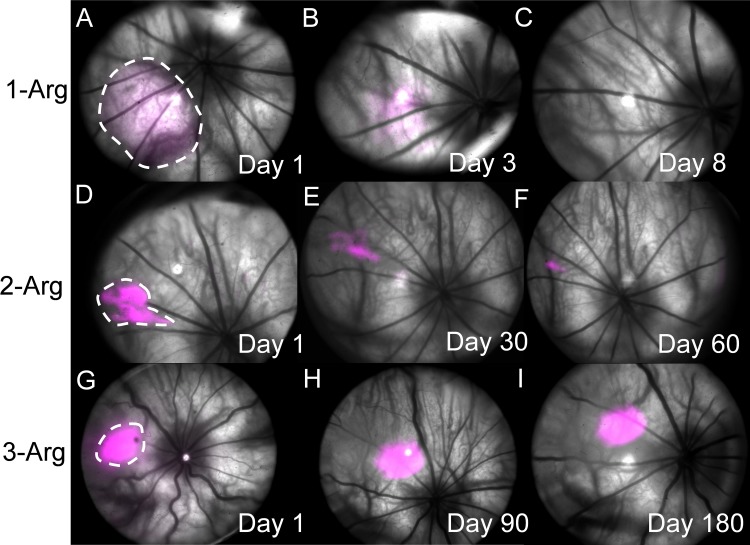
In vivo monitoring of nanoparticle carrier diffusion in rat eyes. (**A**–**C**) Days 1, 3, and 8 after injection of 1-Arg conjugates. (**D**–**F**) Days 1, 30, and 60 after injection of 2-Arg conjugates. (**G**–**I**) Days 1, 90, and 180 after injection of 3-Arg conjugates. Fluorescence images were pseudo-colored in magenta and were overlaid onto fundus images.

Sample images of the nanoparticle fluorescent maps overlaid with fundus images are shown in Figure 3 (see Supplementary Materials for fluorescence images from each animal). The fluorescence intensities were uniformly normalized against the peak intensity of fluorescence on day 1 of 3-Arg nanoparticle injection. As shown in Figures 3A to 3C, the 1-Arg nanoparticles spread into a 6-mm^2^ area in 1 day after injection, and 90% of fluorescence signal diminished by day 8. The 2-Arg nanoparticles (Figs. 3D–F) spread into a 1.7-mm^2^ area, and the fluorescence was detectable up to 2 months. The 3-Arg nanoparticles (Figs. 3G–I) spread to 1.3 mm^2^, and the fluorescence signal was only slightly reduced after 6 months. The behavior of 4-Arg nanoparticles (not shown) is similar to 3-Arg nanoparticles. By comparing the fluorescence locations determined by retinal vasculature landmarks, we found that both 2-Arg and 3-Arg nanoparticles stayed approximately at the original sites, which is in a practical range for targeting lesions therapeutically. However, the slight changes in location shown in the images may be attributed to the imaging angle and ocular orientation when the fundus images were acquired.

**Figure 4:**
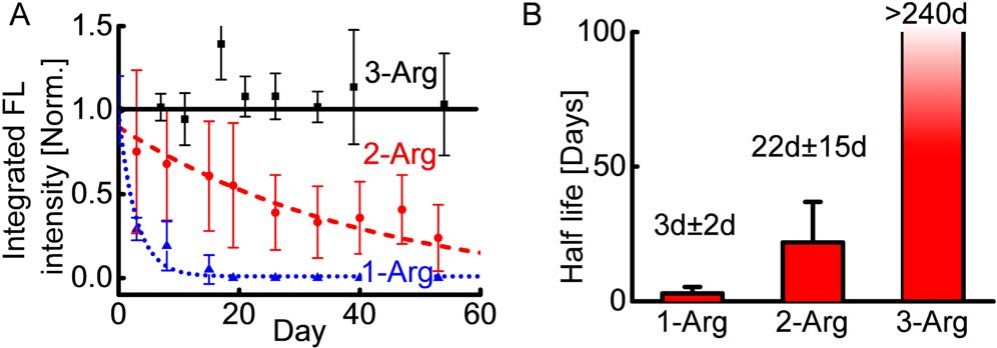
In vivo half-life estimation of nanoparticle carriers. (**A**) Integrated fluorescence intensity of 1-Arg (*n* = 6), 2-Arg (*n* = 5), and 3-Arg (*n* = 3) as a function of time. *Solid black line*, *dashed red line*, and *dash dot blue line* are the corresponding exponential decay fittings. (**B**) Half-lives of nanoparticle carriers estimated from the (**A**). *Error bar* represents 95% confidence interval. The half-life for 3-Arg is more than 240 days. The monitoring was stopped for terminal histological toxicity examination.

We estimated the half-life of nanoparticles from the decays of integrated fluorescence intensities. The relative total amount of nanoparticles was estimated by integrating the fluorescence intensity over the image. The distinct fluorescence intensity decay curves of the three types of nanoparticles in the first 60 days are shown in Figure 4A.

Many factors could collectively affect the intensities of excitation light on fundus and measured fluorescence intensity, including ocular orientation, pupil dilation, and eyelid opening (e.g., with pupil rotated to the canthus, both excitation light and fluorescence light will be blocked by eyelids, resulting in a smaller fluorescence intensity compared with the intensity when the pupil is at the center of the eyelid). Ocular orientation could be potentially controlled by eyeball sucking. Such operation, however, might introduce extraocular pressures and influence the nanoparticle diffusion. During eye imaging, to search the region of interest on the retina while avoiding touching the eyeball, we adjusted the rat position to change the relative system-ocular orientation. The inconsistency of eyeball orientations was minimized by averaging multiple images from different eye orientations and averaging between different animals receiving injections of the same nanoparticles.

The measured in vivo half-lives are shown in Figure 4B. The half-life of 1-Arg nanoparticles was very short (1 to 5 days), which was comparable to that of some peptides reported in rabbit and human vitreous.^14^ The 2-Arg nanoparticles had a moderate half-life (7 to 37 days), thus it could extend the interval to the subsequent injection by approximately 1 additional month. The error bars (confidence intervals) of 1-Arg and 2-Arg nanoparticles were relatively large. This may be attributed to the small sample size and individual physiological differences between animals. We did not obtain the half-life of 3-Arg nanoparticles because all animals receiving these nanoparticle injections showed strong fluorescence at the 240th day, a time at which the animals were euthanized, thus suggesting the half-life of 3-Arg nanoparticles in the vitreous is more than 240 days.

### Histological Evaluations

**Figure 5:**
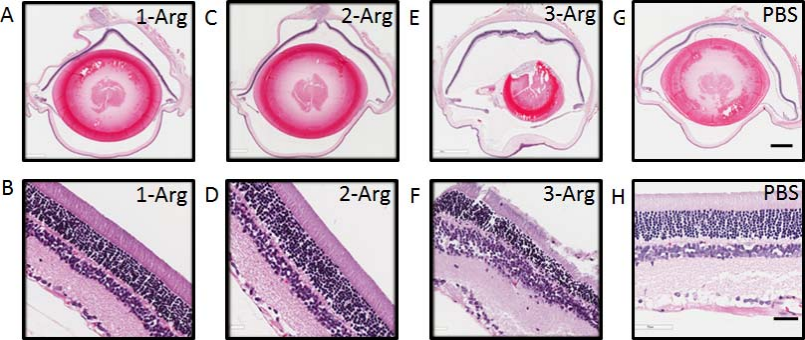
Representative histological images after in vivo monitoring. (**A**, **C**, **E**, **G**) are whole eye sections with injections of 1-Arg, 2-Arg, 3-Arg, and PBS (control group), respectively. *Scale bar*: 1 mm. (**B**, **D**, **F**, **H**) are the corresponding retinal areas. *Scale bar*: 50 μm.

Following the termination of the experiments, animals receiving various nanoparticles for different durations were euthanized and their eyes were subjected to histological evaluation. Representative histological images are shown in Figure 5. We observed no adverse effects on the integrity of ocular structure or infiltration of inflammatory cells. The integrity of the retina was evident with minimal impact of treatments on organization and cellularity of retinal layers. We also observed no sign of infiltrating inflammatory cells, either in the retina or vitreous of eyes form animals from all the groups. The sustained integrity of structure and cellularity of the retina also supports the minimal effect of treatment on the retinal light response function.

## Discussion

Here we demonstrated the extended residence of surface modified nanoparticles in vitreous ex vivo and in vivo. We confirmed that such reduced diffusion originated from the ionic interactions in the vitreous, which is consistent with the earlier reports in which fluorescent tagged albumin was shown to diffuse much slower through vitreous when its acidic residues were converted to cationic charges, and cationic 200-nm polystyrene particles were strongly immobilized.^12,21^ One piece of evidence for ionic binding is that the diffusion of nanoparticles was monotonically reduced with increasing zeta potentials, suggesting that increased binding strength is related to high surface ionization. Additional evidence comes from the observation that in a competitive binding experiment, ionic binding between protamine and HA increased the rate of nanoparticle diffusion. This can be explained by the fact that our nanoparticles anchored themselves to HA by multiple weak ionic interactions. Competitive binding by polycationic protamine occupied the HA, enabling the increased diffusion of nanoparticles.

The biocompatibility of the polycationic particles is critical to the possible clinical applications of our approach. To reduce the total toxicity, we used nano-scale particle carriers and low-toxicity L-Arg cationic groups for anchoring. One advantage of this approach is that small particle size enables minimized adverse effects. Because particle anchoring no longer relies on entrapment within the vitreous fiber network, the drug carriers could be smaller than the mesh of collagen fibers, allowing submicron sterile filtration and thus lessen the intraocular burden, with lower carrier residual aggregation on the retina, and reduced likelihood of immune inflammatory response. Another unique effort we made to reduce toxicity is the use of L-Arg as cationic appended peptides. The other cited uses of L-Arg–based particle/liposome delivery had arginine incorporated into polymer structure or inserted as lipid additional compounds. As a source of cationic charge, L-Arg is considered much safer than all the other cations tested in Ref. 23. It is likely that cells are able to detoxify L-Arg through the charge-eliminating action of peptide deiminases, for which the type 2 enzyme predominates in the retina.^46^ The very slow carrier diffusion from vitreous to retina may also enable ample time for an enzymatic detoxification for lower toxicity. We observed no adverse effects on ocular structural integrity or infiltration of inflammatory cells in the histological examination of rat eyes with three types of our nanoparticles, suggesting little or no ocular toxicity. Further studies will be necessary to better understand the pharmacokinetics of L-Arg labeled carriers in vitreous. For example, L-Arg residues can be converted to citrulline by cells, a common posttranslational event in proteins.^47,48^ This might also happen in our nanoparticle-appended L-Arg peptides in vitreous in a slow manner, as indicated by very long in vivo residence of 3-Arg conjugates.

The nanoparticles not reaching retina could have exited through the trabecular drainage meshwork (TM). Our nanoparticles are small enough that they should not be trapped by TM's cable-like structures.^49,50^ TM contains HA that may ionically trap our nanoparticles; however, the HA concentration in TM is no higher than that in vitreous, which will not further slow down the diffusion rate of nanoparticles. Also, when nanoparticles are close enough to TM cells, nanoparticle charges could be more efficiently neutralized through conversion of arginine to citrulline by enzymatic reactions, accelerating the outflow of the nanoparticles.^46,47^ Also, dextrans have been used clinically over years and are considered safe and readily eliminated via kidney excretion.^51^ Thus, it is likely that CDEX particles also will be excreted after they exit the eye. Confirmations of nanoparticle elimination from vitreous and excretion from the body are needed to further understand long-term toxicity.

The half-life of the nanoparticles in the vitreous was controlled by adjusting the zeta potential. Zeta potential characterizes the extent of surface ionization, which determines the binding strength, diffusion rate, and half-life of nanoparticles. There are several ways to control zeta potential: one is to link peptides with different numbers of positively charged groups. Currently, three discrete zeta potentials of 0.07, 2.4, and 4.9 mV resulted in half-lives of 3 days, 22 days, and more than 8 months, respectively. To adjust half-life, we can also vary the number of peptides on each nanoparticle. The current average number is 22.5 peptides per particle. We could reduce the average loading number of 3-Arg peptides or increase 2-Arg peptides to continuously modulate the zeta potential.^21^ By varying the above parameters, it should be possible to design drug carriers with a wide range of half-lives to fit multiple intraocular delivery objectives for different injection interval requirements.

One limitation of this work is that we did not study the drug delivery in the vitreous, instead we focused on the nanoparticle carriers. The traditional method of embedding drugs into nanoparticles is not applicable because the carriers described here are compact and do not contain a central aqueous phase or large pores. A more suitable strategy could be linking drugs to the surface of nanoparticles with cleavable esters and cross-linkers; for example, Santi et al.^52^ reported that *β*-eliminative cleaving linkers used to conjugate the therapeutic agents and carriers could achieve a drug release half-life in blood up to 1 year. With prolonged anchoring of nanoparticles in vitreous, those linkers will gradually break and smoothly release drug molecules to access the lesion.

Another limitation is that the diffusion rates and half-lives of the same nanoparticles in human vitreous from diseased eyes could be different from those in rat vitreous because of their different vitreous volumes, viscosity, and HA composition. Our injected volume is 2.7% of the rat vitreous, which is comparable to the amount of 1.3% to 2.5% in clinical intravitreal injections.^8,14,53^ However, the anchoring of our nanoparticles relies on HA molecules. Large absolute amounts of nanoparticles may exceed the capacity of local HA, causing different diffusion rates in different areas. Also, human vitreous viscosity varies greatly among individuals; for example, the viscosity can decrease by at least 2-fold in geriatric eyes, which might significantly increase the rate of diffusion,^54^ thus requiring an increased L-Arg content, as in a 4-Arg peptide or with more peptides per particle. In the future, injection of a greater number of nanoparticles into larger vitreous volume (e.g., in rabbit vitreous or bovine vitreous) will be performed and compared with the results in rats. The anchoring in elder donors' vitreous with less HA also will be studied.

## Conclusions

In this work, we demonstrated a novel type of surface modified nanoparticles for sustained drug release in vitreous. The carriers immobilize themselves in vitreous through ionic binding to HA. This strategy significantly increases the carriers' half-life without additional adverse effects on the eye. We experimentally confirmed the ionic binding between nanoparticles and HA by competing binding experiments. We observed reduced diffusion rates of our fabricated nanoparticles ex vivo and increased half-lives in vivo in the rat. The longest clearance period of these nanoparticles in vivo is 240 days. Although such prolonged residence may be attributable to a more highly conjugated subset of nanoparticles with zeta potentials higher than the average, the possibility of very long-term delivery is evident. Histological examinations confirmed no adverse effects on ocular morphology and organization from the third week to the thirty-sixth week. These results demonstrate the potential for this new type of peptide carriers toward sustained therapeutic release against posterior eye diseases.

## Supplementary Material

Supplement 1

## References

[1] National Eye Institute. Prevalence of adult vision impairment and age-related eye diseases in America. Available at: https://nei.nih.gov/eyedata/adultvision_usa. Accessed September 27, 2017.

[2] Antonetti DA, Klein R, Gardner TW. Diabetic retinopathy. *N Engl J Med*. 2012; 366: 1227–1239. 22455417 10.1056/NEJMra1005073

[3] Jager RD, Mieler WF, Miller JW. Age-related macular degeneration. *N Engl J Med*. 2008; 358: 2606–2617. 18550876 10.1056/NEJMra0801537

[4] Kompella UB, Lee VH. Barriers to drug transport in ocular epithelia. : Amidon GL, Topp EM, Lee PI, *Transport Processes in Pharmaceutical Systems*. New York: Marcel Dekker; 1999: 317–376.

[5] Gaudana R, Ananthula HK, Parenky A, Mitra AK. Ocular drug delivery. *AAPS J*. 2010; 12: 348–360. 20437123 10.1208/s12248-010-9183-3 PMC2895432

[6] Del Amo EM, Rimpelä A-K, Heikkinen E, Pharmacokinetic aspects of retinal drug delivery. *Prog Retin Eye Res*. 2017; 57: 134–185. 28028001 10.1016/j.preteyeres.2016.12.001

[7] Fagan XJ, Al-Qureshi S. Intravitreal injections: a review of the evidence for best practice. *Clin Exp Ophthalmol*. 2013; 41: 500–507. 23078366 10.1111/ceo.12026

[8] Federici TJ. Intravitreal injections. : Stern GA, ed. *Focal Points: Clinical Modules for Ophthalmologists*. San Francisco, CA: American Academy of Ophthalmology; 2009.

[9] Falavarjani KG, Nguyen QD. Adverse events and complications associated with intravitreal injection of anti-VEGF agents: a review of literature. *Eye*. 2013; 27: 787–794. 23722722 10.1038/eye.2013.107 PMC3709385

[10] Gibson JM, McGinnigle S. Diabetes: intravitreous ranibizumab for proliferative diabetic retinopathy. *Nat Rev Endocrinol*. 2016; 12: 130–131. 26794438 10.1038/nrendo.2016.1

[11] Williams GA. IVT injections: health policy implications. *Review of Ophthalmology*. 2014; 21: 62–64.

[12] Xu QG, Boylan NJ, Suk JS, Nanoparticle diffusion in, and microrheology of, the bovine vitreous ex vivo. *J Control Release*. 2013; 167: 76–84. 23369761 10.1016/j.jconrel.2013.01.018 PMC3693951

[13] Shmueli RB, Ohnaka M, Miki A, Long-term suppression of ocular neovascularization by intraocular injection of biodegradable polymeric particles containing a serpin-derived peptide. *Biomaterials*. 2013; 34: 7544–7551. 23849876 10.1016/j.biomaterials.2013.06.044 PMC3838902

[14] Genetech, Inc. *Prescribing Information for Lucentis*. South San Francisco, CA: Genetech; 2017.

[15] Rosenfeld PJ, Brown DM, Heier JS, Ranibizumab for neovascular age-related macular degeneration. *N Engl J Med*. 2006; 355: 1419–1431. 17021318 10.1056/NEJMoa054481

[16] Jager RD, Aiello LP, Patel SC, Cunningham ETJ. Risks of intravitreous injection: a comprehensive review. *Retina*. 2004; 24: 676–698. 15492621 10.1097/00006982-200410000-00002

[17] Schwartz SG, Scott IU, Flynn HW, Stewart MW. Drug delivery techniques for treating age-related macular degeneration. *Expert Opin Drug Deliv*. 2014; 11: 61–68. 24219407 10.1517/17425247.2013.859135

[18] Sakurai E, Ozeki H, Kunou N, Ogura Y. Effect of particle size of polymeric nanospheres on intravitreal kinetics. *Ophthalmic Res*. 2001; 33: 31–36. 11114602 10.1159/000055638

[19] Hsu J. Drug delivery methods for posterior segment disease. *Curr Opin Ophthalmol*. 2007; 18: 235–239. 17435432 10.1097/ICU.0b013e3281108000

[20] Bourges JL, Gautier SE, Delie F, Ocular drug delivery targeting the retina and retinal pigment epithelium using polylactide nanoparticles. *Invest Ophthalmol Vis Sci*. 2003; 44: 3562–3569. 12882808 10.1167/iovs.02-1068

[21] Kim H, Robinson SB, Csaky KG. Investigating the movement of intravitreal human serum albumin nanoparticles in the vitreous and retina. *Pharm Res*. 2009; 26: 329–337. 18958405 10.1007/s11095-008-9745-6

[22] Kim N, Jiang D, Jacobi AM, Synthesis and characterization of mannosylated pegylated polyethylenimine as a carrier for sirna. *Int J Pharm*. 2012; 427: 123–133. 21864664 10.1016/j.ijpharm.2011.08.014 PMC3237934

[23] Zern BJ, Chu H, Osunkoya AO, Gao J, Wang Y. A biocompatible arginine-based polycation. *Adv Funct Mater*. 2011; 21: 434–440. 23393493 10.1002/adfm.201000969 PMC3564668

[24] Veiseh O, Kievit FM, Liu V, In vivo safety evaluation of polyarginine coated magnetic nanovectors. *Mol Pharm*. 2013; 10: 4099–4106. 24099143 10.1021/mp4005468 PMC3946456

[25] Sarker SR, Aoshima Y, Hokama R, Inoue T, Sou K, Takeoka S. Arginine-based cationic liposomes for efficient in vitro plasmid DNA delivery with low cytotoxicity. *Int J Nanomedicine*. 2013; 8: 1361. 23630419 10.2147/IJN.S38903 PMC3626367

[26] Liu C, Liu X, Rocchi P, Qu F, Iovanna JL, Peng L. Arginine-terminated generation 4 pamam dendrimer as an effective nanovector for functional sirna delivery in vitro and in vivo. *Bioconjug Chem*. 2014; 25: 521–532. 24494983 10.1021/bc4005156

[27] Zinderman CE, Landow L, Wise RP. Anaphylactoid reactions to dextran 40 and 70: reports to the United States Food and Drug Administration, 1969 to 2004. *J Vasc Surg*. 2006; 43: 1004–1009. 16678697 10.1016/j.jvs.2006.01.006

[28] Jones CI, Payne DA, Hayes PD, The antithrombotic effect of dextran-40 in man is due to enhanced fibrinolysis in vivo. *J Vasc Surg*. 2008; 48: 715–722. 18572351 10.1016/j.jvs.2008.04.008

[29] US Food and Drug Administration. Select Committee on GRAS Substances (SCOGS) Opinion: Dextran. 1975 Available at: https://www.fda.gov/Food/IngredientsPackagingLabeling/GRAS/SCOGS/default.htm. Accessed September 27, 2017.

[30] Wasiak I, Kulikowska A, Janczewska M, Dextran nanoparticle synthesis and properties. *PLoS One*. 2016; 11: e0146237. 26752182 10.1371/journal.pone.0146237 PMC4713431

[31] Kaewprapan K, Inprakhon P, Marie E, Durand A. Enzymatically degradable nanoparticles of dextran esters as potential drug delivery systems. *Carbohydr Polym*. 2012; 88: 875–881.

[32] Senanayake TH, Warren G, Vinogradov SV. Novel anticancer polymeric conjugates of activated nucleoside analogues. *Bioconjug Chem*. 2011; 22: 1983–1993. 21863885 10.1021/bc200173e PMC3200571

[33] Chau Y, Padera RF, Dang NM, Langer R. Antitumor efficacy of a novel polymer-peptide-drug conjugate in human tumor xenograft models. *Int J Cancer*. 2006; 118: 1519–1526. 16187287 10.1002/ijc.21495

[34] Susa M, Iyer AK, Ryu K, Doxorubicin loaded polymeric nanoparticulate delivery system to overcome drug resistance in osteosarcoma. *BMC Cancer*. 2009; 9: 399. 19917123 10.1186/1471-2407-9-399 PMC2788581

[35] Ghosh AK, Brindisi M. Organic carbamates in drug design and medicinal chemistry. *J Med Chem*. 2015; 58: 2895–2940. 25565044 10.1021/jm501371s PMC4393377

[36] Park D, Wu W, Wang Y. A functionalizable reverse thermal gel based on a polyurethane/peg block copolymer. *Biomaterials*. 2011; 32: 777–786. 20937526 10.1016/j.biomaterials.2010.09.044 PMC2991555

[37] Mishra A, Seethamraju K, Delaney J, Willoughby P, Faust R. Long-term in vitro hydrolytic stability of thermoplastic polyurethanes. *J Biomed Mater Res A*. 2015; 103: 3798–3806. 26097127 10.1002/jbm.a.35523

[38] Tang Y, Labow R, Santerre J. Enzyme induced biodegradation of polycarbonate-polyurethanes: dose dependence effect of cholesterol esterase. *Biomaterials*. 2003; 24: 2003–2011. 12628819 10.1016/s0142-9612(02)00563-x

[39] Le Goff MM, Bishop PN. Adult vitreous structure and postnatal changes. *Eye*. 2008; 22: 1214–1222. 18309340 10.1038/eye.2008.21

[40] Chaudhuri A, Hallett PE, Parker JA. Aspheric curvatures, refractive indices and chromatic aberration for the rat eye. *Vision Res*. 1983; 23: 1351–1363. 6666037 10.1016/0042-6989(83)90146-3

[41] Crank J. The Mathematics of Diffusion. Oxford, United Kingdom: Oxford University Press; 1979.

[42] Berkowitz BA, Lukaszew RA, Mullins CM, Penn JS. Impaired hyaloidal circulation function and uncoordinated ocular growth patterns in experimental retinopathy of prematurity. *Invest Ophthalmol Vis Sci*. 1998; 39: 391–396. 9477999

[43] Li H, Liu W, Zhang HF. Investigating the influence of chromatic aberration and optical illumination bandwidth on fundus imaging in rats. *J Biomed Opt*. 2015; 20: 106010. 26502233 10.1117/1.JBO.20.10.106010 PMC4881312

[44] Liu W, Li H, Shah RS, Simultaneous optical coherence tomography angiography and fluorescein angiography in rodents with normal retina and laser-induced choroidal neovascularization. *Opt Lett*. 2015; 40: 5782–5785. 26670511 10.1364/OL.40.005782 PMC6711669

[45] Delori FC, Webb RH, Sliney DH. Maximum permissible exposures for ocular safety (ANSI 2000), with emphasis on ophthalmic devices. *J Opt Soc Am A*. 2007; 24: 1250–1265. 10.1364/josaa.24.001250 17429471

[46] Bonilha VL, Shadrach KG, Rayborn ME, Retinal deimination and pad2 levels in retinas from donors with age-related macular degeneration (AMD). *Exp Eye Res*. 2013; 111: 71–78. 23562679 10.1016/j.exer.2013.03.017 PMC3683981

[47] Bhattacharya SK. Retinal deimination in aging and disease. *IUBMB Life*. 2009; 61: 504–509. 19391158 10.1002/iub.184

[48] Algeciras ME, Takahara H, Bhattacharya SK. Mechanical stretching elevates peptidyl arginine deiminase 2 expression in astrocytes. *Curr Eye Res*. 2008; 33: 994–1001. 19085382 10.1080/02713680802447113 PMC3998668

[49] Sun YY, Keller KE. Hyaluronan cable formation by ocular trabecular meshwork cells. *Exp Eye Res*. 2015; 139: 97–107. 26247678 10.1016/j.exer.2015.07.018 PMC4573334

[50] Du Y, Roh DS, Mann MM, Funderburgh ML, Funderburgh JL, Schuman JS. Multipotent stem cells from trabecular meshwork become phagocytic TM cells. *Invest Ophthalmol Vis Sci*. 2012; 53: 1566–1575. 22297497 10.1167/iovs.11-9134 PMC3339918

[51] Mehvar R, Shepard TL. Molecular-weight-dependent pharmacokinetics of fluorescein-labeled dextrans in rats. *J Pharm Sci*. 1992; 81: 908–912. 1279158 10.1002/jps.2600810914

[52] Santi DV, Schneider EL, Reid R, Robinson L, Ashley GW. Predictable and tunable half-life extension of therapeutic agents by controlled chemical release from macromolecular conjugates. *Proc Natl Acad Sci U S A*. 2012; 109: 6211–6216. 22474378 10.1073/pnas.1117147109 PMC3341049

[53] Bakri SJ, Cameron JD, McCannel CA, Pulido JS, Marler RJ. Absence of histologic retinal toxicity of intravitreal bevacizumab in a rabbit model. *Am J Ophthalmol*. 2006; 142: 162–164. 16815270 10.1016/j.ajo.2006.03.058

[54] Balazs EA. The vitreous. *Int Ophthalmol Clin*. 1973; 13: 169–187. 4204768

