# Supplementary material for: Sustaining Intravitreal Residence With L-Arginine Peptide-Conjugated Nanocarriers
Source: Invest Ophthalmol Vis Sci. 2017 Oct;58(12):5142–50. doi: 10.1167/iovs.17-22160 (PMC5634351; doi:10.1167/iovs.17-22160)
Supplement: Supplement 1 [file IOVS-17-22160-s01.pdf]

Sustaining intravitreal residence with L-Arginine peptide-conjugated nanocarriers

Hao Li[1], Wenzhong Liu[1], Christine M. Sorenson[2], Nader Sheibani[3], Daniel M. Albert[4], Thulani Senanayake[5], Serguei Vinogradov[5], Jack Henkin[6,*] Hao F. Zhang[1,7,*]

1. Department of Biomedical Engineering, Northwestern University, 2145 Sheridan Rd., Evanston, IL, 60208, USA
2. Department of Pediatrics, University of Wisconsin-Madison, WI 53705
3. Departments of Ophthalmology & Visual Sciences, Biomedical Engineering, and Cell and Regenerative Biology, University of Wisconsin-Madison, WI 53705
4. Department of Ophthalmology, Casey Eye Institute, Oregon Health Sciences University, Portland, OR, 97239
5. Department of Pharmaceutical Sciences, College of Pharmacy University of Nebraska Medical Center, Omaha, NE 68198
6. Center for Developmental Therapeutics, Northwestern University, Evanston IL, 60208
7. Department of Ophthalmology, Northwestern University, Chicago IL, 60611

* Please send all correspondences regarding nanoparticle and chemical synthesis to Jack Henkin (j-henkin@northwestern.edu) and all correspondences regarding imaging to Hao F. Zhang (hfzhang@northwestern.edu).

Supplementary information

1. Nanoparticle carrier synthesis

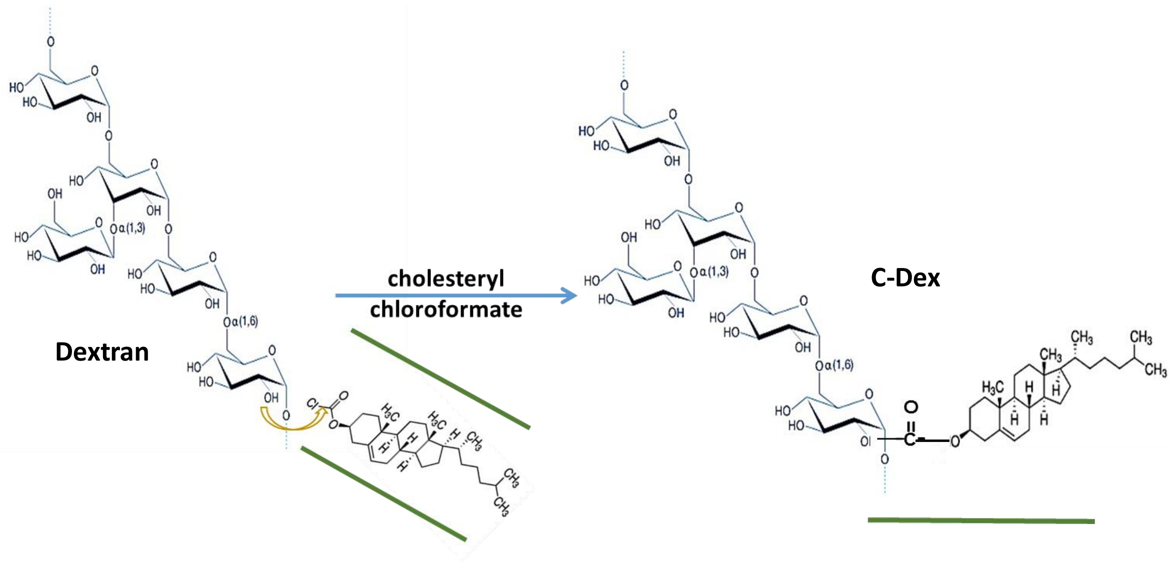

Scheme S1. Cholesterol modification of dextran to form CDEX.

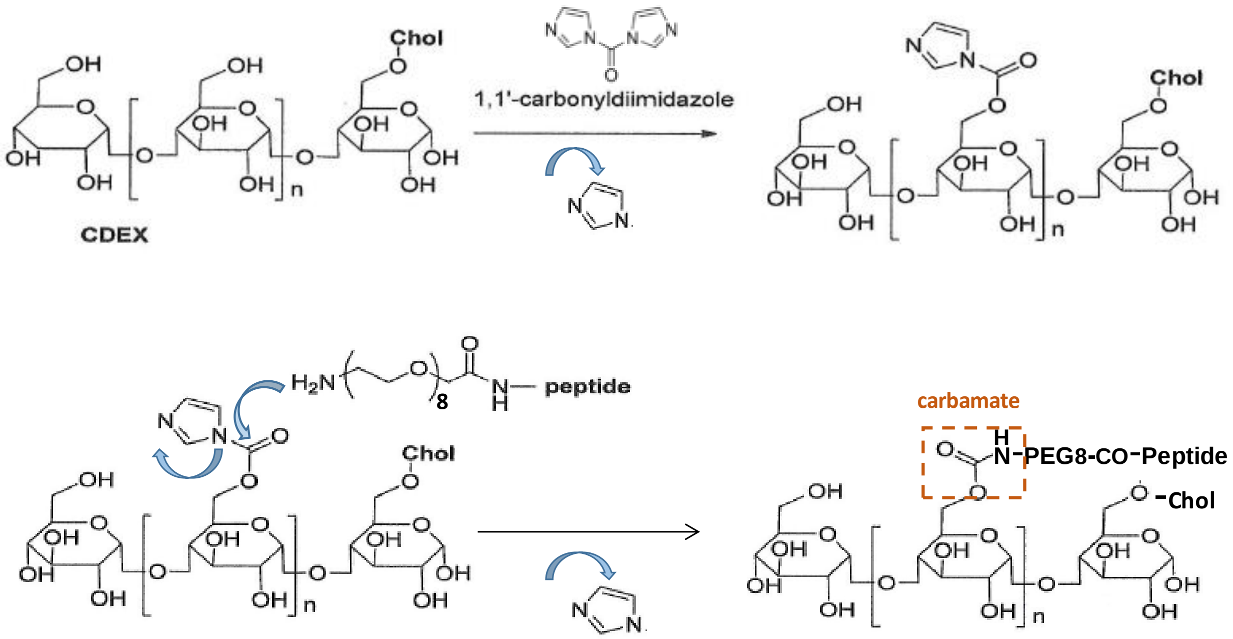

Scheme S2. Two-step carbamate linkage of amino-PEG peptides to CDEX. Carbamate bond is highlighted in the lower-right structure.

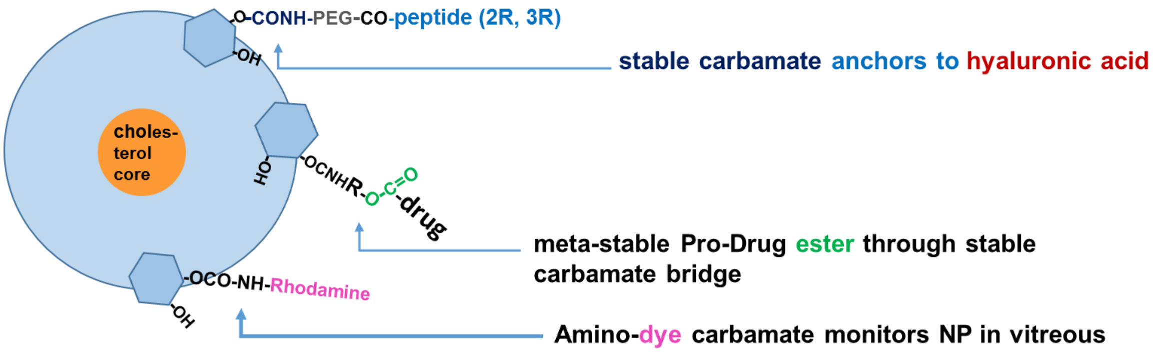

Scheme S3. Types of carbamate conjugates to CDEX. Stable attachment is accomplished for anchoring amino-PEG peptides (top) and for amino-dyes (lower). When the nitrogen atom of the carbamate is from an esterified amino alcohol, the ester bridge (center) is metastable and may release drug through non-catalyzed hydrolysis. The latter may also be a peptide or protein. NP: nanoparticle.

2. *Ex vivo* diffusion rate estimation

2.1 Mathematical model

As illustrated in Fig. S1A, rat vitreous body is in a crescent shape and could be roughly considered as a two-dimensional structure [1]. Assuming right after injection, the drug is uniformly distributed in a circle with a diameter of *h*, the concentration distribution in cylindrical coordinates is [2]

$$C = \frac{1}{2} C_0 \left(\operatorname{erf} \frac{h-x}{2\sqrt{Dt}} + \operatorname{erf} \frac{h+x}{2\sqrt{Dt}} \right), \tag{1}$$

where x is the distance from the origin; t is time; C is the concentration at location x at time t; C_0 is the initial concentration; D is the diffusion rate and erf is the error function.

We used relative area changes to calculate diffusion rates. We define the diffusion area is within the boundary where the concentration dropping to e^{-1} of the 95% of the peak concentration. Based on Eq. (1), the normalized concentration distribution and diffusion area is determined by t, D and h. Therefore, a series of diffusion area under different time could be used to numerically calculate D and h. Experimentally, once a time

sequence of fluorescence images were captured, the fluorescence emitting area in each image was calculated within the boundary, where the fluorescence intensity dropping to e^{-1} of the 95% of the peak intensity. We used the measured sizes of fluorescence emitting areas and corresponding time point to calculate *D* using Least Squares Fit according to Eq. (1), assuming fluorescence intensity is proportional to the drug concentration.

2.2 Ex vivo *nanoparticle diffusion experimental measurement*

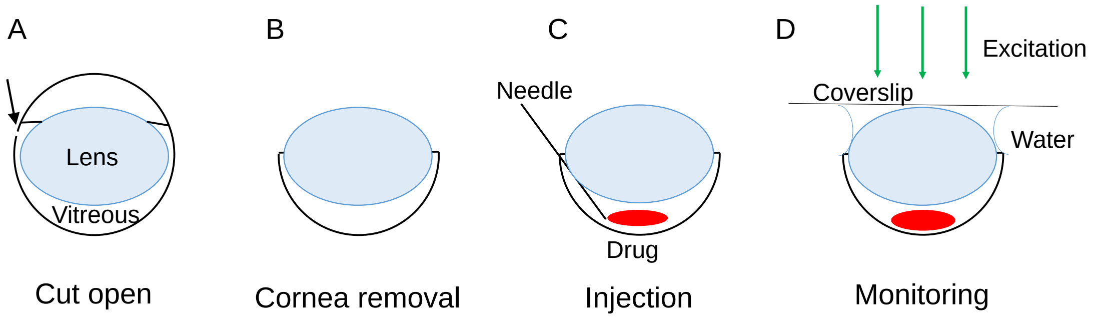

Figure S1. Illustration of *ex vivo* diffusion rate estimation. We made a small incision at the corneal limbus using a scalpel blade (**A**) (labelled by the black arrow), circumferentially cut along the limbus, and removed the cornea and iris (**B**). 1.5 μL of Rhodamine labelled nanoparticle colloidal gel was injected to the center of vitreous by a microliter syringe (**C**). A coverslip was added on top of the opened eyeball with water filled in between. The dye labelled nanoparticles were excited by a 532-nm CW laser and the fluorescence was monitored to estimate the nanoparticle diffusion (**D**).

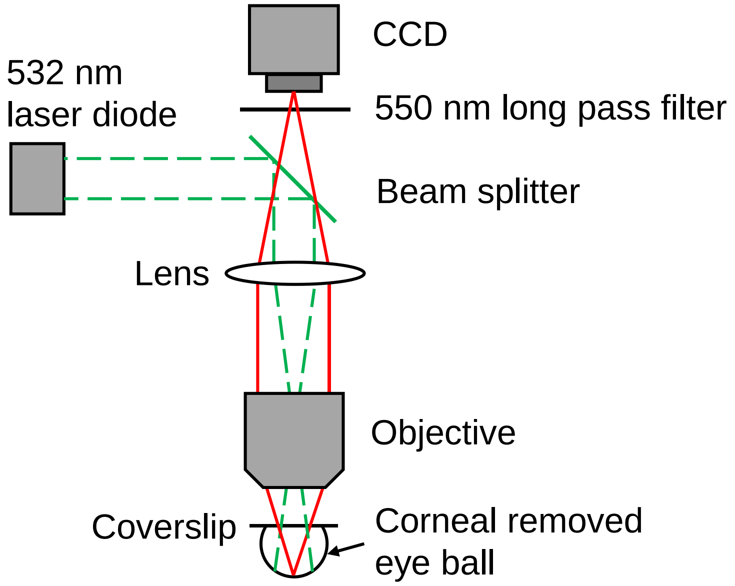

Figure S2. Schematic of the optical setup for *ex vivo* fluorescence monitoring. The opened eyeball with the coverslip was placed under a stereo microscope (SMZ1500, Nikon, simplified as a combination of objective and a lens). Excitation light (Continuous wave 532 nm laser, 10 mW) was combined into the illumination light path using a beam splitter. Fluorescence images were captured using a high-sensitive CCD (PixelFly qe, PCO AG, Germany) equipped on the imaging port of the microscope and a long pass optical filter (FEL0550, Thorlabs; cut-off wavelength: 550 nm).

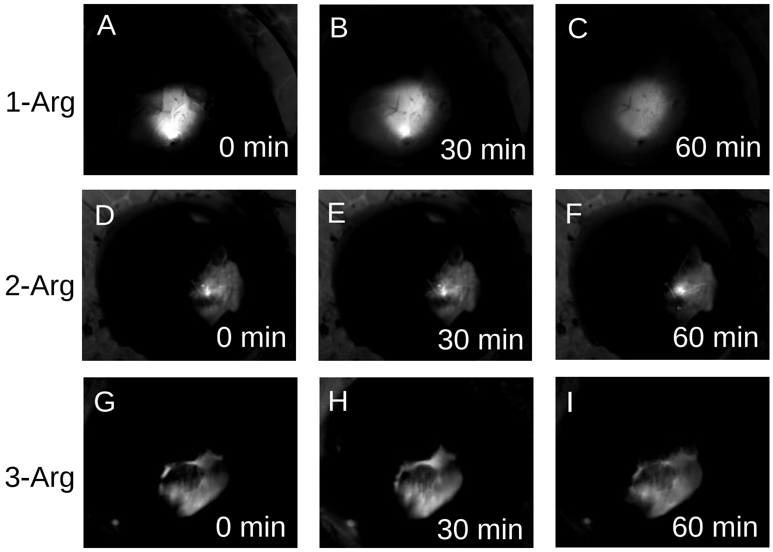

Figure S3. Examples of fluorescence image sequence in *ex vivo* diffusion rate estimation.

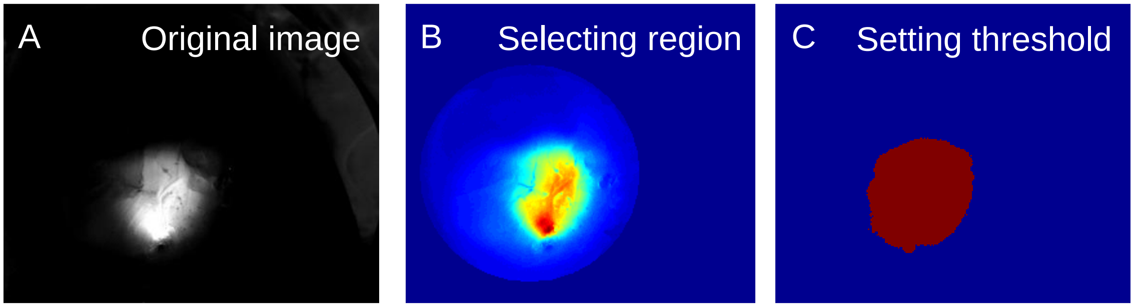

Figure S4. Diffusion rate estimation procedure. The original fluorescence image **A** was first normalized by 95% of the maximum pixel intensity and cropped to the eye region (**B**). The cropped image was binarized by the intensity threshold of e^{-1}, and the fluorescence area was then calculated by counting the non-zero pixel numbers (**C**).

2.3. Competitive binding to hyaluronic acid between nanoparticles and protamine

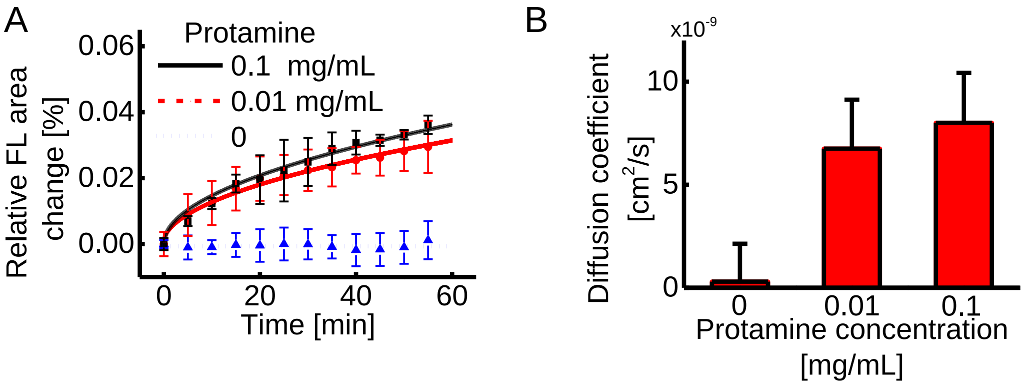

Figure S5. *Ex vivo* results of competitive binding to hyaluronic acid between nanoparticles and protamine. Protamine with different concentrations were mixed with 3-Arg nanoparticle solution (0.4 mg/mL) and injected into rat eyeball vitreous. The corresponding fluorescence was monitored in one hour. **A** Relative fluorescence area change as a function of time. **B** Diffusion coefficient estimated from A. FL: fluorescence.

3. *In vivo* nanoparticle half-life measurement in rat vitreous

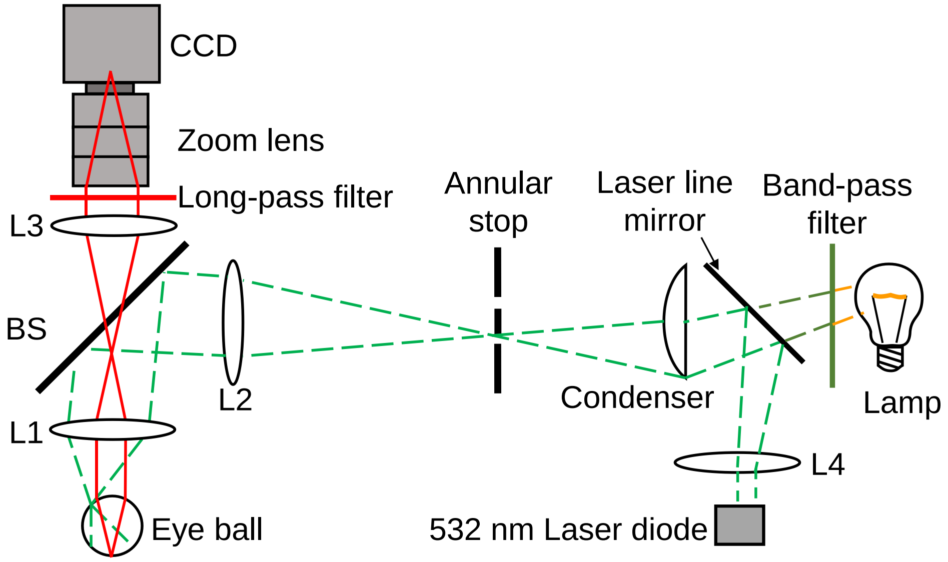

Figure S6. Schematic of the fundus camera for reflectance and fluorescence imaging of living rat eyes. L1~L4: achromatic lenses with the focal lengths of 25 mm, 60 mm, 50 mm and 30 mm, respectively (Thorlabs). Zoom lens: 12–36 mm, F# 1:2.8 (Computar). Condenser: 20 mm focal length with diffuser on the plano side (Thorlabs). BS: 50:50 beam splitter. Laser line mirror: 45 degree, 532 nm central wavelength. Light source: 100 W halogen lamp. Band-pass filter: 12.7 nm bandwidth, 580 nm center wavelength (Edmund Optics). Long-pass filter: 550 nm cut-on wavelength (Edmund Optics). The illuminating powers for reflectance imaging and florescence imaging were 0.2 mW and 0.25 mW, respectively. The exposure times were 0.2 s for reflectance imaging and 2 s for fluorescence imaging.

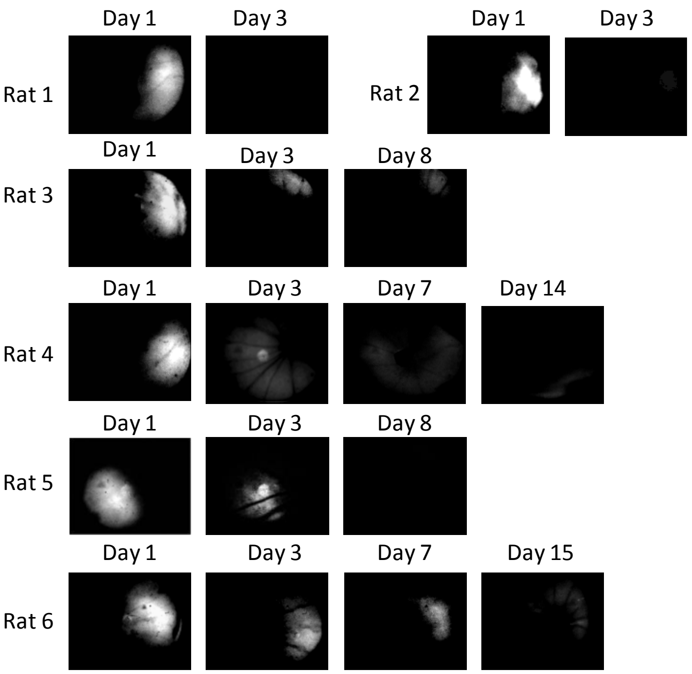

Figure S7. Fluorescence intensity evolutions of the rats (N=6) receiving injection of 1-Arg conjugate.

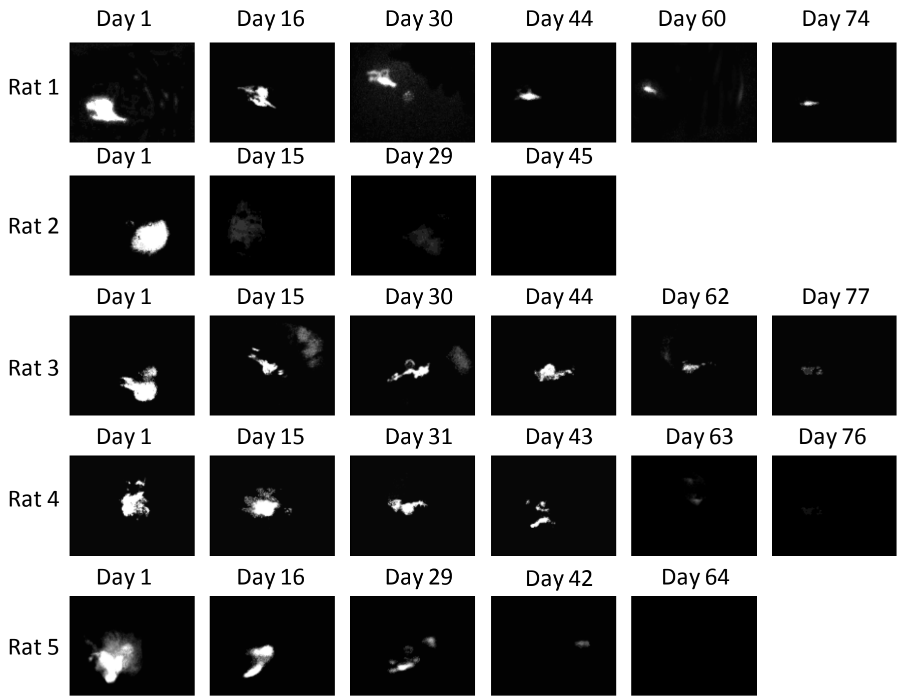

Figure S8. Fluorescence intensity evolutions of the rats (N=5) receiving injection of 2-Arg conjugate.

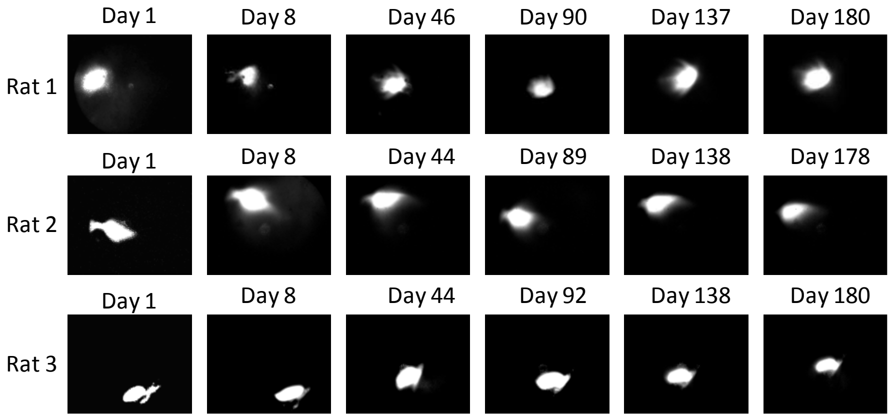

Figure S9. Fluorescence intensity evolutions of the rats (N=3) receiving injection of 3-Arg conjugate.

Reference

[1] A. Chaudhuri, P.E. Hallett, J.A. Parker, Aspheric curvatures, refractive indices and chromatic aberration for the rat eye, Vision Res, 23 (1983) 1351-1363.
[2] J. Crank, The mathematics of diffusion, Oxford university press, 1979.
